# Thermal Degradation and Carbonization Mechanism of Fe−Based Metal−Organic Frameworks onto Flame−Retardant Polyethylene Terephthalate

**DOI:** 10.3390/polym15010224

**Published:** 2023-01-01

**Authors:** Tianyi Ma, Wenqing Wang, Rui Wang

**Affiliations:** 1 Materials Design & Engineering Department, Beijing Institute of Fashion Technology, Beijing 100029, China; 2 Beijing Key Laboratory of Clothing Materials R&D and Assessment, Beijing Engineering Research Center of Textile Nanofiber, Beijing Institute of Fashion Technology, Beijing 100029, China

**Keywords:** metal-organic framework, polyester, flame-retardant materials, thermal degradation, carbonization

## Abstract

Currently, the metal-organic framework (MOF) is a promising candidate for flame-retardant polymers. In this study, a Fe-based MOF, MIL-88B(Fe), was introduced to polyethylene terephthalate (PET) and 3-hydroxyphenylphosphinyl-propanoic acid copolymer (P-PET) to reduce the fire hazard involved in using PET. The limiting oxygen indexes (LOIs) of MIL-PET and MIL-P-PET improved by 27% and 30%, respectively. The UL-94 level achieved for MIL-P-PET was V-0 rating. The thermal degradation and carbonization mechanisms of MIL-PET and MIL-P-PET were systematically investigated through thermogravimetric analysis coupled with a Fourier transform infrared spectroscopy (TG-IR), pyrolysis-gas chromatography-mass spectrometry (Py-GC-MS), X-ray photoelectron spectroscopy (XPS), and Raman spectrum combined with quantum chemical molecular dynamics simulation. With the addition of MIL-88B(Fe), high graphitization and a hard flammability char residual were generated. Compared with neat PET, the ferric ions efficiently catalyzed the homolytic cleavage and dehydrogenation of PET to produce a large amount of CO_2_ and terephthalic acid for MIL-PET in gas phase. Rough and hierarchical char residual with ferric oxide was also generated when temperatures exceeded 600 °C. However, the carbonization process was inhibited due to the coordinated complex between phosphorus and ferric ions in MIL-P-PET, invaliding the decarboxylation and generating more benzoic acid and its precursor, which led to heavy smoke.

## 1. Introduction

Over the past few years, polyethylene terephthalate (PET) has become one of the most widely used polymers in textiles, film, and plastic products due to its good thermal and chemical stability, high mechanical strength, low permeability to gas, and excellent spinnability [1]. However, the high flammability of pristine PET restricts its broad application to fields in which fire risk and fire hazard are a main concern. Among the current flame retardants available, metal flame retardants, such as aluminum hydroxide [2], magnesium hydroxide [3], metal oxide [4,5], and other metal ions [6], are still excellent flame-retardant candidates, with the advantages of low cost as well as high efficiency. Primarily, metal complexes, which generally contain organic ligands, especially including flame-retardant elements, endow materials with more flame-retardant properties and easy compatibility with polymer matrixes. These have attracted great interest and show great potential in fire resistant polymer synthesis.

By virtue of their structural periodicity, ordered porosity, and rich functionality, metal-organic frameworks (MOFs) are commonly used in catalysis [7,8], medical industries [9,10], hydrogen storage [11], the adsorption of carbon dioxide [12,13], etc. The various metal centers containing MOFs, including nickel (Ni), cobalt (Co), iron (Fe), zinc (Zn), and copper (Cu) [14,15,16,17,18], could efficiently catalyze char formation. The porosity of MOFs would not make them a means of gas storage in the fire condition but could also increase the specific surface area of metal ions to develop the catalytic performance. Furthermore, due to their stable coordination and covalent bonds, most MOFs are thermally and chemically stable, making them a feasible option for polymer composite processing.

Recently, the synergistic use of a variety of flame retardants with MOFs showed superior flame retardancy, displaying, for instance, reduced graphene oxide [19], MoS_2_ [20], and layered double hydroxides [21]. To our knowledge, flame retardants containing phosphorus are the most widely applied and commercialized flame retardants for PET, and the addition of both phosphorus flame retardants and MOFs into polymers showed excellent fire proofing capability. Hou et al. [22] synthesized a Co-MOF blended with 6H-dibenz(C,E)(1,2) oxaphosphorin-6-oxide (DOPO), for which the peak heat release rate (PHRR) and smoke production rate (SPR) decreased by 27% and 56%, respectively. Huang et al. [23] synthesized a nitrogen and phosphorus ionic liquid functional MIL-101-NH_2_ and blended it in the epoxy to improve fire safety. Cai et al. [24] made DOPO react with the –C=N– of methylimidazole in a Zn-MOF to raise the flame-retardant quality of epoxy resin (EP). Therefore, in this work, ferric MOFs combined with phosphorous flame retardants were introduced to reduce the flammability of PET.

Generally, the thermal degradation and carbonization mechanisms of flame retardants and polymer composites are significant for revealing the underlying flame retardancy process. The generation of char residue with phosphorus flame retardants is considered to be an essential part in MOFs’ condensed phase flame-retardant mechanism in the polymer matrix [16,22,23,25,26,27]. Wang et al. [28] proved that a Co-MOF with APP was beneficial for generating a dense char residue to protect the inner matrix. However, according to Zhang’s research, ferric ions might be significantly antagonistic to DOPO because of possible decreasing radical scavenging activity [29]. Therefore, it is essential to detect the interaction between phosphorus flame retardant and MOFs in PETs and their combustion and flame-resistant properties.

Except for in the current experimental study, which included FTIR, TG, SEM, and BET [30,31,32], quantum chemical molecular dynamics simulation is a promising tool to investigate the thermal degradation and carbonization of various MOF composites. Shigemoto [33] et al. employed quantum chemistry to study the thermal degradation mechanisms of Sb, Ge, Ti, and Zn complexes. They concluded that β hydrogen abstraction by the alkoxy ligand of catalyst reaction was the primary step in thermal degradation. In our previous study, quantum chemistry calculation was a reliable method for predicting the flame retardancy of different cationic MOFs, which were verified by experimental data [34]. However, detailed research on the mechanisms of thermal oxide degradation of MOFs and phosphorus-containing flame-retardant PET composites has not yet been reported.

Herein, MIL-88B(Fe), in which the terephthalic acid (TPA) ligand was the same monomer as in PET, was in-suit blended into the PET and 3-hydroxyphenyl-phosphinyl propanoic acid (CEPPA)-PET, which were named MIL-PET and MIL-P-PET, respectively. The flammability, as well as the forced combustion, of MIL-PET was investigated via the limiting oxygen index (LOI), vertical combustion test (VFT), micro cone calorimetry (MCC), and cone calorimetry (CC) test. The thermal and thermal oxide degradation behaviors were characterized using thermal gravimetric analysis (TGA), Fourier infrared spectroscopy (FTIR), pyrolysis mass spectrometry (Py-GC-MS), thermogravimetric analysis coupled to Fourier transform infrared spectroscopy (TG-IR), and Raman spectrometry combined with quantum chemistry molecular dynamics in different atmospheres. This research provided new insight into the thermal degradation and carbonization mechanisms of Fe-based MOFs and phosphorus synergistic flame-retardant PET.

## 2. Materials and Methods

### 2.1. Materials

N,N-dimethylformamide (DMF, 99.8%), Terephthalic acid (TPA, 99%), and Ferric nitrate nonahydrate (Fe(NO_3_)_3_·9H_2_O, 95%) were all purchased from J&K Scientific Ltd. (Beijing, China). ethanol (EtOH, 95%) was purchased from Beijing Tongguang Co., and 3-hydroxyphenyl-phosphinyl propanoic acid (CEPPA) was provided by Zhenbei Co., Ltd. (Hangzhou, China). Pure terephthalic acid (PTA, industrial grade) and Ethylene glycol (EG, industrial grade) were supplied by the Sinopec Group (Tianjin, China). Triphenyl phosphite (TPI, analytical pure) and Diantimony trioxide (Sb_2_O_3_, analytical pure) were purchased from Macklin Biochemical Co., Ltd. (Shanghai, China). HyMax 1010 (1010, industrial grade) was provided by Langyi Functional Materials Co., Ltd. (Shanghai, China). All chemicals were used directly without further purification.

### 2.2. Preparation of MIL-88B(Fe)

MIL-88B(Fe) was synthesized through a solvothermal reaction according to previous paper research [35]. Measures of 1.66 g (1.0 mmol) TPA and 4.04 g (1.0 mmol) Fe(NO_3_)_3_·9H_2_O were added to 100.0 mL DMF. After being magnetically stirred at 400 rpm for 30 min at room temperature, the mixed solution was poured into the Teflon lining of a stainless steel autoclave and heated at 140 °C for 24 h in the oven. The product was centrifuged at 10,000 rpm for 10 h, washed three times with ethanol, and dried at 120 °C for 12 h in a vacuum. MIL-88B(Fe) was obtained after the product had naturally cooled down to room temperature.

### 2.3. Preparation of MIL-88B(Fe) and PET Composites

Firstly, MIL-88B(Fe) was added to 314.0 g EG and underwent ultrasonic dispersion for 20 min. Then, 700.0 g TPA, 0.10 g Sb_2_O_3_ (as the catalyst), and MIL-88B(Fe) in EG were added into the polymerizer. The mixture was then heated to about 270 °C under a nitrogen atmosphere to esterify and discharge water. Measures of 30.0 g EG, 0.70 g 1010 (an anti-hydrolytic agent), and 0.35 g TPI were subsequently replenished to the polymerizer under atmospheric pressure for 30 min. Finally, PET was obtained in a high vacuum at 270 °C for about 2–3 h, after which the mechanical stirring reached the specified torque. Neat PET, 0.2MIL-PET, 0.6MIL-PET, 1.0MIL-PET, P-PET, 0.2MIL-P-PET, and 0.6MIL-P-PET composites were also synthesized using a similar method, except for the addition of 4.5 phr CEPPA in the esterification procedure. The samples’ names and formulas are listed in Table 1.

### 2.4. Characterizations

Wide-angle X-ray diffraction (WAXD) of samples were acquired through Co radiation in a D8 ADVANCE (Bruke, Bremen, Germany) at a scanning rate of 2°/min and with a scan range of 5 to 45°, at 40 kV and 15 mA. Fourier transform infrared spectrometry (FTIR) was conducted using a Nicolet 6700 FTIR spectrometer (Thermo Fisher Scientific, New York, NY, USA) in ATR mode. The spectra were collected in the optical range of 400–4000 cm^−1^, with a scanning number of 64. Thermogravimetric analysis (TGA) was conducted using a Thermal analyzer (TG 209, Netzsch, Waldkraiburg, Germany) in nitrogen and air atmospheres, and the gas flow rate was 100 mL/min. The samples were kept within the range of 5 to 10 mg and then heated from 30 °C to 700 °C at a ramp rate of 10 °C/min. The limiting oxygen index (LOI) test was carried out using an Oxygen Index Apparatus (Dynisco, Franklin, TN, USA) according to ASTM D2863-2008, and the sample size was 100.0 × 6.5 × 3.0 mm^3^. The vertical flame test (UL-94) was conducted using the GZF-3 Horizontal and Vertical Combustion Meter (Nanjing Jiang Ning Analytical Instrument Co., Ltd., Nanjing, China) in reference to ASTM D3801, and the sample size was 130.0 × 13.0 × 3.0 mm^3^. The cone calorimetry (CC) test was conducted according to ISO 5660-1 using the fire testing technology (FTT) standard cone calorimeter (FTT, Hong Kong, China) under an external heat flux of 50 kW/m^2^, and the samples were tailored to 100.0 × 100.0 × 3.0 mm^3^. Thermogravimetric analyses coupled to Fourier transform infrared spectroscopy (TG-IR) curves were obtained by the Mettler Toledo TGA2 coupled with an iS50 FTIR spectrophotometer (Thermo Fisher Scientific, New York, NY, USA), heated from room temperature to 800 °C with a linear heating rate of 20 °C/min under a nitrogen flow of 100 mL/min with a resolution of 32 cm^−1^. Pyrolysis coupled with a gas chromatography-mass spectrometry (Py-GC-MS) test was carried out to identify the gaseous products. Firstly, the sample was treated in an EGA/PY-3030 pyrolyzer (Frontier, Tokyo, Japan) in a helium atmosphere by heating from an ambient temperature to 700 °C at a rate of 200 °C/s, after which it was pyrolyzed for 300 s. Afterwards, the products were carried through helium to the DB-5MS capillary column, wherein the temperature of the capillary column (30.0 × 0.25 × 0.25 m^3^) of GC was firstly held at 50 °C for 2 min and then increased to 250 °C at a heating rate of 20 °C/min and kept at 250 °C for 20 min. The MS indicator was operated in the electron impact mode with an electron energy level of 70 eV and a scanning range of 20–650 m/z. A Raman spectroscopy was applied to characterize the graphitization degree of residual char. This was conducted using the RM2000 Laser Raman microscope (Renishaw, London, UK). The sample was thoroughly dried before the test, and the laser wavelength was 325 nm. X-ray photoelectron spectroscopy (XPS) analysis was carried out with a thermo escalab 250 Xi (Thermo Fisher Scientific, New York, NY, USA) equipped with an Al Kα radiation source. The Olympus Bx51 carried out the optical microscopy in optical reflection mode. A scanning electron microscope (SEM) was acquired in the JSM-7500F (JEOL, Tokyo, Japan). Temperature measurements of the samples in combustion were collected via a K-type thermal couple, as shown in Figure S1.

### 2.5. Quantum Chemistry Calculation

Geometric optimization and quantum chemistry molecular dynamics at 727 °C (1000 K) and 1527 °C (1800 K) for the unit of PET and its composition were calculated using the GFN1-xTB [36,37] method, which was carried by xtb-6.4.1. Structures were generated by Multiwfn [38].

## 3. Results and Discussion

### 3.1. Characterization of MIL-88B(Fe) and Its PET Composites

**Figure 1:**
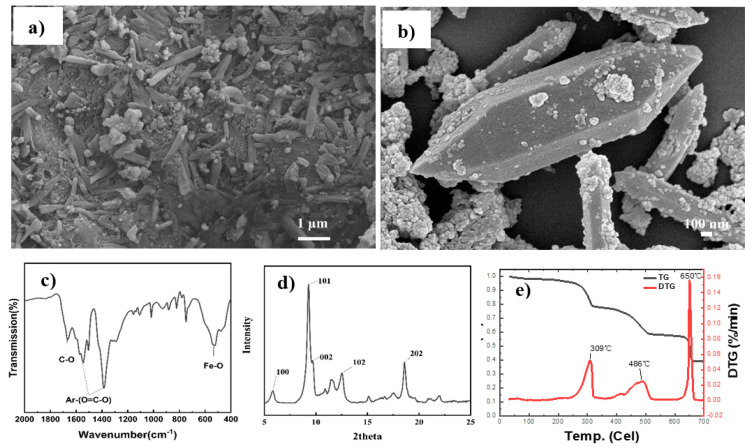
(**a**,**b**) SEM images, (**c**) FTIR spectrum, (**d**) XRD pattern and (**e**) TG/DTG curves for MIL-88B(Fe).

The micro-morphology and constitution of synthesized MIL-88B(Fe) were confirmed through SEM, FTIR, and XRD. As seen in Figure 1b, crystals with a rod-like shaped and high dispersibility were also observed with a uniform size of about 10 µm in length and 1 µm in diameter. The FTIR spectrum of MIL-88B(Fe) is given in Figure 1c to identify the functional groups. The two sharp adsorption peaks at 1540 cm^−1^ and 1396 cm^−1^ were attributed to asymmetric (νas(C–O)) and symmetric (νas(C–O)) vibrations of carboxyl groups, respectively. C–H bending vibrations of benzene were observed at 749 cm^−1^ while a peak at 538 cm^−1^ was ascribed to the Fe-O vibrations. The reflection patterns of the XRD spectra of the as-prepared materials given in Figure 1d were consistent with a previous report [34], indicating the successful synthesis of MIL-88B(Fe). Moreover, MIL-88B(Fe) displayed a good thermal stability, with maximum thermal degradation temperatures of 309, 486, and 650 °C as shown in Figure 1e. This makes possible its potential usage in PET synthesis and/or further processing.

**Figure 2:**
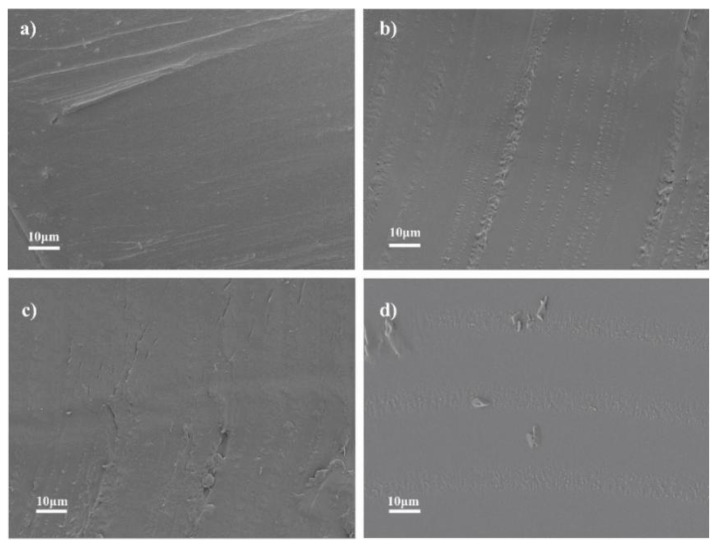
SEM images of (**a**) PET, (**b**) 0.6MIL-PET, (**c**) P-PET, (**d**) 0.6MIL-P-PET.

The morphologies of the MIL-88B(Fe) and PET composites, including 0.6MIL-PET, P-PET, and 0.6MIL-P-PET composites, are given in Figure 2. As can be seen in Figure 2, all the MIL-88B(Fe) and PET composites had smooth cross-sections, and ferric, carbon, and oxygen elements were all homogeneously distributed in the PET matrix (Figure S2), suggesting that MIL-88B(Fe) had good compatibility with the PET matrix without evident aggregates in the PET or P-PET copolymer within experimental addition ratio.

### 3.2. Thermal Stability

**Figure 3:**
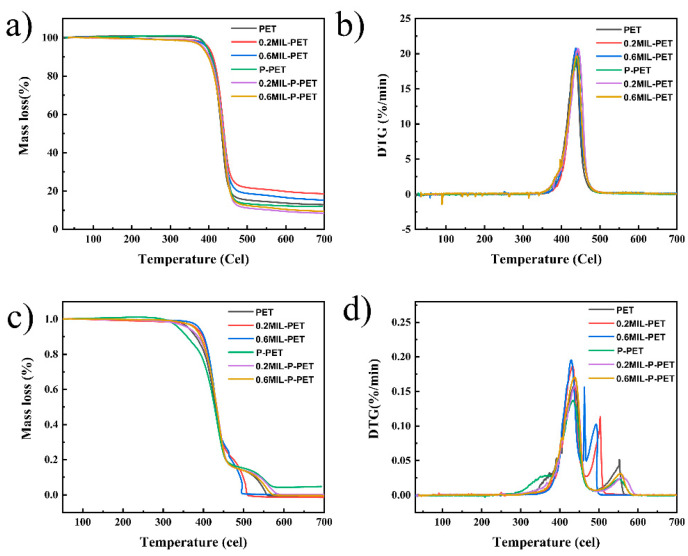
(**a**) TG (thermogravimetric analysis) in N_2_ atmosphere (**b**) DTG (differential thermal gravity) in N_2_ atmosphere (**c**) TG in air atmosphere (**d**) DTG in air atmosphere of PET, 0.2MIL-PET, 0.6MIL-PET, P-PET, 0.2MIL-P-PET, 0.6MIL-P-PET.

The TG and DTG (differential TG) curves of PET, 0.2MIL-PET, 0.6MIL-PET, P-PET, 0.2MIL-P-PET, and 0.6MIL-P-PET are demonstrated in Figure 3. The values of *T*_5%_ (temperature of 5% mass loss), *T*_max_ (temperature of maximal rate mass loss), and char residue at 700 °C in different atmospheres are listed in Table 2. From Figure 3a, it can be seen that the value of *T*_5%_ for 0.2MIL-PET was delayed by 4 °C compared with PET in an N_2_ atmosphere, which suggested that a small amount of MIL-88B(Fe) postponed the thermal decomposition reaction. This regular trend was also observed in the *T*_max_ change. Nevertheless, when the MIL-88B(Fe) addition kept increasing, *T*_5%_ was decreased back to pristine PET. Moreover, it was found that MIL-88B(Fe) would increase the content of char residue, which composed of PET degradation or cross-linking products and ferric complex at 700 °C in an N_2_ atmosphere. Conversely, the MIL-88B(Fe) blending in the P-PET copolymer exhibited a different rule to the MIL-PET composites. The char residue of MIL-P-PET gradually reduced after adding more MIL-88B(Fe) with delayed values of *T*_5%_ and *T*_max_, which indicated that MIL-P-PET might effectively accelerate the thermal pyrolysis of PET in a lower temperature and with inhibited char generation.

TG and DTG curves of PET and its composites in air condition are displayed in Figure 3c,d. The results obtained in an air atmosphere were different from those in the N_2_ atmosphere, with one additional thermal degradation step. Specifically, in the first degradation step, the onset temperature of degradation (*T*_5%_) in P-PET advanced by about 30 °C, while MIL-P-PET had a similar *T*_5%_ value to PET. This indicated that the accelerated decomposition mechanism of P-PET might be invalid for the MIL-P-PET series. Similarly, the second thermo-oxidative degradation peaks of the MIL-P-PET series also illustrated a different pattern for P-PET, which advanced by about 100 °C and had an increased peak value. This two-step decomposition process in an air atmosphere was also observed in polycarbonate (PC) [39,40], epoxy resin (EP) [41], and aromatic polyurethane (PU) [42,43], while this was not observed for polylactic (PLA) [44], poly(methyl methacrylate) (PMMA) [45], or neat polystyrene (PS) [46]. This might be explained by the benzene structure in the matrix, and the thermal oxidation decomposition of benzene rings could be remarkably changed by MIL-88B(Fe). However, the effect of MIL-88B(Fe) was weakened in the MIL-P-PET series. Meanwhile, the residue of MIL-P-PET dropped to zero, suggesting the inhibition of char residue formation.

### 3.3. Flammability of MIL-PET and MIL-P-PET Composites

The flammability of each of the MIL-PET composites were assessed via VFT and LOI tests, and the results are presented in Table 3. The neat PET was flammable, with a LOI value of 22%, and exhibited severe smoldering phenomenon (t1 and t2 total 58.4 s), which in turn accelerated combustion and would reduce the chance of human escape in the case of a fire. With the MIL-88B(Fe) addition, the value of LOI for MIL-PET experienced a slight increase of 27%. Moreover, the average burning time was shortened, proving the efficient fire resistance of MIL-88B(Fe) in PET. However, the UL-94 classification was still a V-2 rating due to severe melt droplets. As previously reported, CEPPA made outstanding flame retardants, owing to the simultaneous phosphorus-related radicals capture in the gas phase and the char generation in the condensed phase. The LOI values of P-PET and MIL-P-PET increased to 29% and 30%, respectively. Furthermore, the average total burning time significantly declined within 4 s. It is worth noting that after the combination of MIL-88B(Fe) and CEPPA, MIL-P-PET reached a V-0 rating without the presence of melt dripping during combustion, suggesting the synergistic flame-retardant effect of MOF and phosphorus flame retardant.

### 3.4. Forced Combustion of MIL-PET and MIL-P-PET Composites

**Figure 4:**
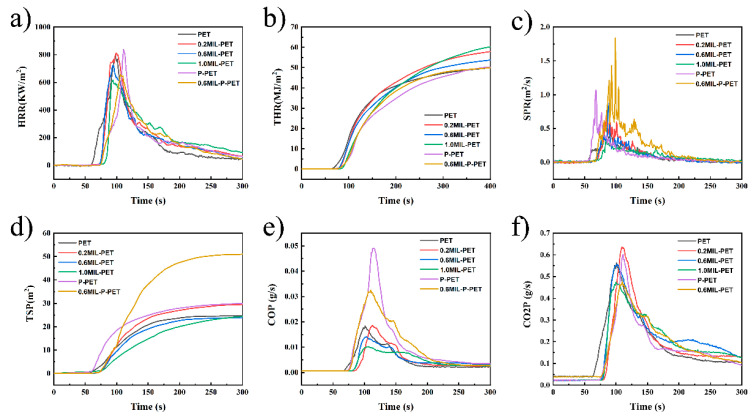
(**a**) Heat release rate (HRR), (**b**) total heat release (THR), (**c**) smoke production rate (SPR), (**d**) total smoke production (TSP), (**e**) carbon monoxide production (COP), and (**f**) carbon dioxide production (CO2P) curves of PET, 0.2MIL-PET, 0.6MIL-PET, P-PET, 0.6MIL-P-PET.

To further investigate the combustion behavior of the MIL-88B(Fe), PET, and P-PET copolymers, a CC test was employed to simulate a developing fire scenario, which was reliable and similar to actual combustion circumstances. The results of the CC test are shown in Figure 4. The heat release rate (HRR), peak heat release rate (pHRR), and total heat release (THR) data would reflect the real combustion exothermic situation. It can be seen that MIL-88B(Fe) was beneficial for the pHRR decrease in PET, with a maximal reduction value of 23.12% in 1.0MIL-PET (Table 4). However, THR gradually increased after the addition of MIL-88B(Fe). These results demonstrated that MIL-88B(Fe) in the PET matrix reduced combustion intensity but catalyzed the continuous thermal-oxidative decomposition of PET to release more heat. Similarly, compared with the P-PET copolymer, the pHRR value of 0.6MIL-P-PET showed a 21.75% decrease. Contrarily, the THR value of 0.6MIL-P-PET compensated for the THR increasing in MIL-PET in the presence of CEPPA. This could be attributed to the different mechanisms of thermal-oxidative degradation in the case of phosphorus flame retardants’ interaction with MIL-88B(Fe).

The value of smoke produce rate (SPR) and total smoke production (TSP) reflected the amount of smoke released during combustion. When the MIL-88B(Fe) addition was increased to 0.6 and 1.0 wt%, both the SPR and TSP of MIL-PET composites reduced compared to pristine PET, as shown in Figure 4c,d. By contrast, blending the same amount of MIL-88B(Fe) in P-PET led to a significant increase in smoke production, and the TSP value of 0.6MIL-P-PET increased by 1.7 times. This could be explained by the interaction of MIL-88B(Fe) and CEPPA, which might promote the decomposition of PET into lighter smoke to generate a nonflammable precursor to the residual char.

The carbon monoxide production (COP) and carbon dioxide production (CO2P) displayed in Figure 3e,f and the values of peak carbon monoxide production (pCOP) and peak carbon dioxide production (pCO2P) given in Table 3 reflected the influence of gas during combustion. As compared with neat PET, the values of pCOP and pCO2P for 1.0MIL-PET decreased by 44.45% and 19.34%, respectively. Similar trends were also observed in MIL-P-PET and P-PET. It was evident that the addition of MIL-88B(Fe) perfectly reduced the composites’ carbon monoxide and carbon dioxide emissions. Additionally, the prolysis product was most likely to be transferred to either smoke emission or condensed char layer.

Generally, a micro cone calorimeter (MCC) is applied to assess the combustion properties of a trace-amount polymer matrix. In this work, MCC was employed as an effective tool to detect the heat release of the polymer in complete thermal oxide degradation. As given in Figure S3, the PET, MIL-PET, P-PET, and MIL-P-PET showed two peaks in their MCC curves, similar to the DTG curves in air atmosphere aforementioned. The heat release of the first step for MIL-PET showed a gradual increase of 2.0%. This might be explained by the violent decomposition, as proved in TG. The initial temperature of the second step advanced by about 90 °C in MIL-PET compared with the others. At the same time, the value of pHRR for the second step sharply increased, while the THR did not change significantly overall. The pHRR of P-PET gradually increased, but on the contrary, MIL-P-PET had less impact on the HRR release curve. This could be explained by the catalytic performance of MIL-88B(Fe) and/or the elimination of the phosphorus’s dehydration effect. Compared to the measurement conditions, the temperature growth demonstrated by the MCC test increased slower than that of the CC test, and it provided more chance for the degradation products to be cross-linked by ferric ions catalysis to generate a stable char residue outside, resulting in a delay to the heat release of inner char residue. The incomplete decomposition of the polymer matrix was the main reason why pHRR in the CC test decreased, but pHRR and THR in the MCC increased. However, under continuous high temperature and thermal radiation in the CC test, more minor parts of the inner char of MIL-PET would still slowly degrade to release heat and smoke after initial intense decomposition, leading to a high value of HRR during the CC test. However, in CC conditions, MIL-88B(Fe) was beneficial to decrease the violent combustion and allow for self-extinguish in a mild condition.

### 3.5. Char Analysis

**Figure 5:**
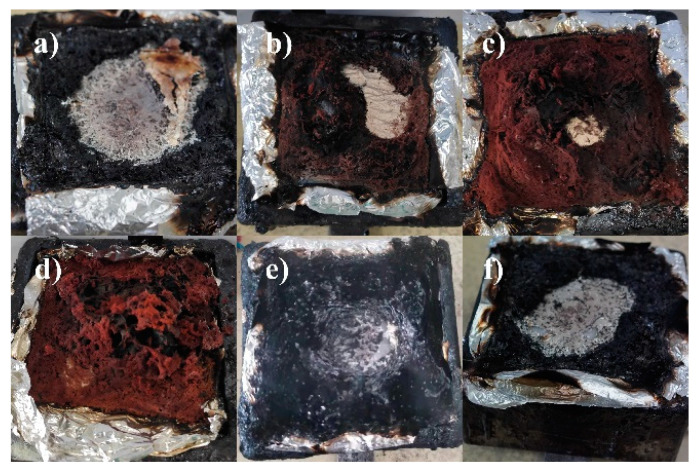
Digital photos of char residue of (**a**) PET, (**b**) 0.2MIL-PET, (**c**) 0.6MIL-PET, (**d**) 1.0MIL-PET, (**e**) P-PET, (**f**) 0.6MIL-P-PET.

**Figure 6:**
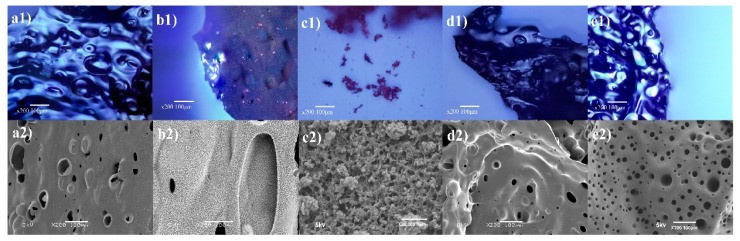
Optical microscope and SEM images of char residue of (**a1**,**a2**) PET, (**b1**,**b2**) MIL-PET, (**c1**,**c2**) crimson floccules in char residue, (**d1**,**d2**) P-PET, (**e1**,**e2**) MIL-P-PET.

To better understand the present char formation of MIL-88B(Fe), the morphology and chemical composition of char residues were systematically detected through SEM, FTIR, Raman, and XPS measurements. Digital photos of the char residue for PET, MIL-PET, P-PET, and MIL-P-PET are shown in Figure 5. It was obvious that the volume of char residue grew larger with the addition of MIL-88B(Fe) to the PET. Meanwhile, crimson floccules appeared on the surfaces of char residue, which was presumed to be ferric oxide, i.e., Fe_2_O_3_. Interestingly, the crimson floccules were not present in the char residue of MIL-P-PET, indicating that ferric ions might be transformed into another complex other than ferric oxide in P-PET combustion. From the SEM images of the char residual as given in Figure 6, there were many tiny pores on the char surface of PET and P-PET, while a rougher powder-like exterior layer existed on the char of MIL-PET with rare pores. After amplification, some crimson dendritic crystals were observed from the crimson floccules of char residue (Figure 6(c2)), providing evidence of the existence of iron oxide in the char of MIL-PET composites.

**Figure 7:**
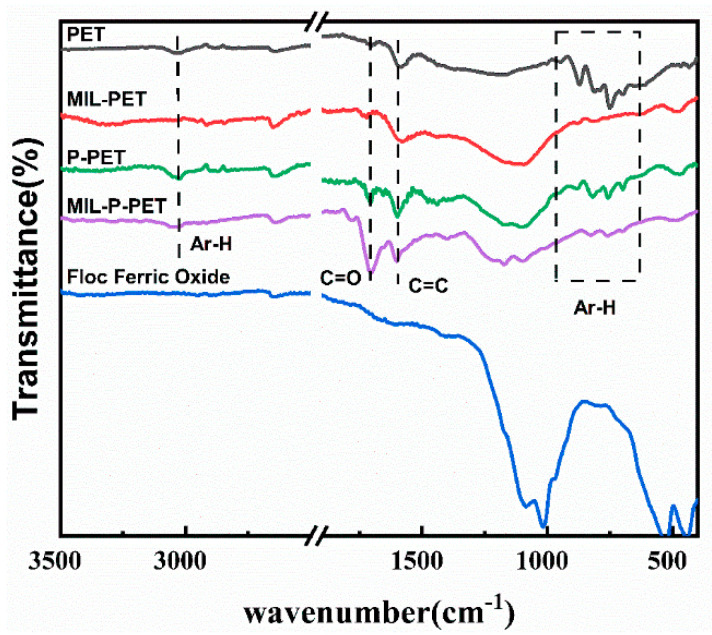
FTIR spectra of the char residue of PET, MIL-PET, P-PET, MIL-P-PET.

The FTIR spectra of char residue are shown in Figure 7. The adsorption peaks of 600–1000 cm^−1^ and 3025 cm^−1^ were assigned to C-H stretch vibration and out-plane vibration of benzene, respectively, in which the latter disappeared after blending MIL-88B(Fe) in the PET. This indicated that iron ions in MIL-PET catalyzed the dehydrogenation of benzene, which was assigned as the second pyrolysis process in TG and MCC. Significantly, the double-peaking in 458 cm^−1^ and 553 cm^−1^ were attributed to Fe-O stretch vibration, providing proof of the presence of ferric oxide in the char of MIL-PET. The C=O stretch vibration of MIL-PET at about 1730 cm^−1^ had a slightly red shift, while the peaks of other char were around 1710 cm^−1^, which demonstrated that the environment of C=O had been altered by the introduction of MIL-88B(Fe). The spectra of MIL-P-PET and P-PET char residue were similar to that of pure PET. However, the transmittance at 1710 cm^−1^ increased a lot, which indicated the concentration of C=O increasing after combustion. Additionally, the amount of C–H in MIL-P-PET decreased, suggesting that ferric ions might have coordinated with the phosphorous compounds and restricted its effect on benzene dehydrogenation. Furthermore, an increase in the number of C–O–C bonds in MIL-PET char was observed in both C1s and O1s XPS spectra (Figure S4). The area ratio between the C=O and C–C of MIL-P-PET increased a lot compared with other samples, proving that MIL-88B(Fe) coordinated with the P-PET copolymer would invalidate the decarboxylation reaction.

Laser Raman spectroscopy is one of the most common procedures for determining the graphitization degree of char residue. In general, the lower area ratio of the D band (or D1 band) at 1350 cm^−1^ and G band at 1590 cm^−1^ (*I*_D_/*I*_G_) had a strong relationship with higher degrees of graphitization. In this study, to further accurately describe the properties of char residue, the D3 band at 1540 cm^−1^ and the D4 band at about 1180 cm^−1^ were also fitted by the Gaussian-Lorentz. To be specific, the D3 band originated from the amorphous sp2-bonded form carbon-like functional group or organic molecules in disordered amorphous carbon, while the D4 band related to the different sp2- and sp3-bonded forms in very poorly organized materials. According to the Sheng Group’s [47] earlier studies, lower *I*_D3_/*I*_G_ and (*I*_D3_ + *I*_D4_)/*I*_G_ could be applied to describe the lower combustion reactivity and higher graphitization degree of a char structure.

**Figure 8:**
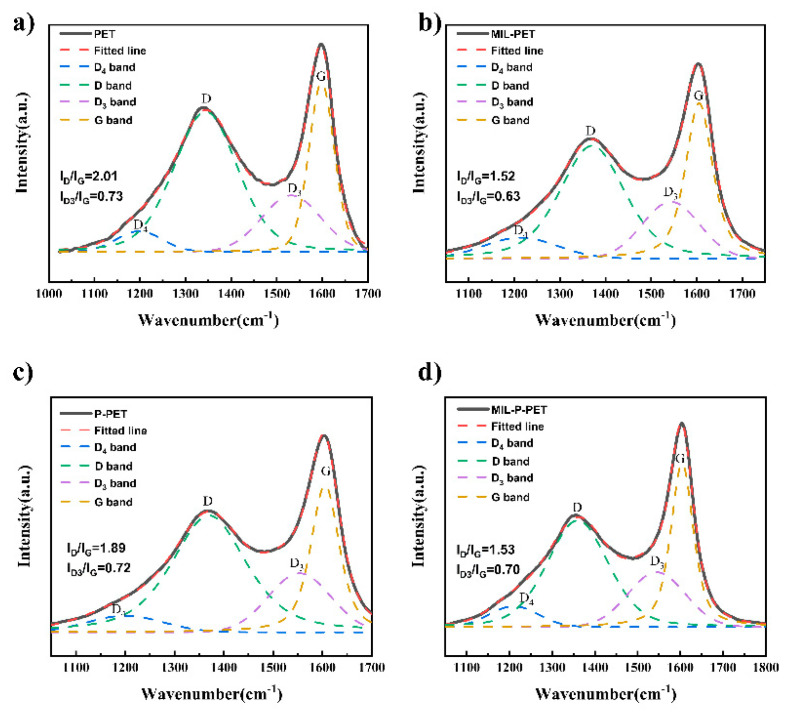
Raman spectra and its fitted lines of the char residue of (**a**) PET, (**b**) MIL-PET, (**c**) P-PET, (**d**) MIL-P-PET.

The key parameters and Raman spectra of different types of char residue are shown in Figure 8 and Table S1. Obviously, the value of *I*_D_/*I*_G_ of the char residue for MIL-PET, P-PET, and MIL-P-PET showed significant decline compared to neat PET. The minimum value of *I*_D_/*I*_G_ was observed in MIL-PET, close to the value of MIL-P-PET. It indicated that ferric ions in PET composites were beneficial to the catalytic formation of high graphitization carbon. Furthermore, the value of *I*_D3_/*I*_G_ of MIL-PET char residue decreased a lot when comparing with other samples, suggesting the hard flammability of the char residue in MIL-PET in which the less functional group existed. The char residue of MIL-P-PET also showed increased flame-retardant properties with the lower *I*_D3_/*I*_G_ and (*I*_D3_ + *I*_D4_)/*I*_G_ values.

**Figure 9:**
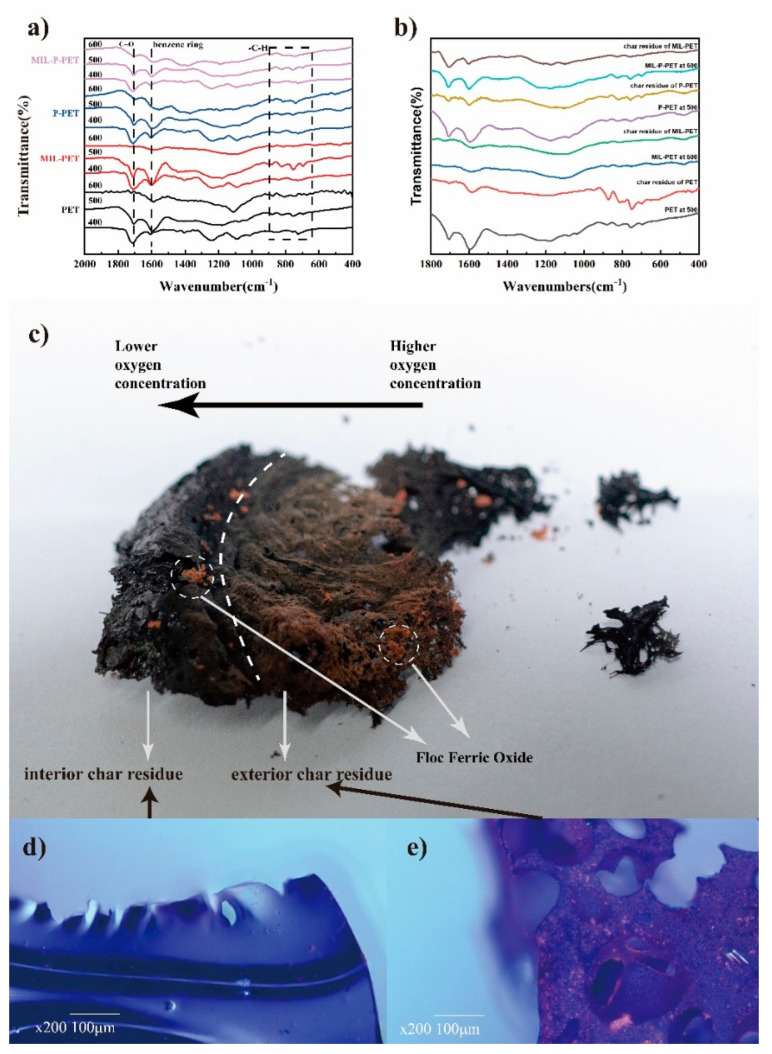
FTIR spectra of the char residue of PET composites at various constant temperatures (**a**). (**b**) Comparison between CC test and muffle furnace. (**c**) Digital photo of MIL-PET at 600 °C. Optical microscope images of (**d**) interior and (**e**) exterior char residue.

To simulate the char formation process, PET, MIL-PET, P-PET, and MIL-P-PET composites were placed in a muffle furnace at 400, 500, and 600 °C, respectively, which was accordant to the onset temperature of the first and second step in the DTG and MCC tests. FTIR and an optical microscope were employed to characterize the char residues and the results are displayed in Figure 9. Figure 9a revealed that with heating temperature increasing, the adsorption peak at 1704 cm^−1^ (C=O) of PET composites gradually disappeared, and the peak at 1595 cm^−1^, i.e., the benzene ring, was significant at lower temperatures. When the temperature reached 500 °C, the peaks of benzene ring vibration at 1595 cm^−1^ were more obvious than C=O stretch vibration at 1704 cm^−1^ due to the decarboxylation reaction of the polymer matrix. The dehydrogenation reaction of MIL-PET decreased, while other PET composites were still evident after the temperature reached 600 °C (peaks between 600 and 900 cm^−1^). Moreover, the adsorption peaks attributed to C–O–C vibration became wider, indicating different environmental C–O–C bonds generated with temperature increases. The FTIR spectra of char residue in the CC test and the muffle furnace simulation experimental are compared in Figure 9b. The structures of the char residue of PET, P-PET, and MIL-P-PET at a constant 500 °C were similar to the char residue from the CC test. However, the char residue structure of MIL-PET exhibited similarity to CC char only after the continual heating temperature exceeded 600 °C. Moreover, the optical images of PET composites char residue in the muffle furnace were given in Figure S5. This further proved that the rough char residue of MIL-PET was mainly generated in the second pyrolysis step when the temperature exceed 600 °C, owing to the ferric ions’ catalysis.

Figure 9c shows a digital photo of the char residue at 600 °C and uncovered the generation procedure of MIL-PET char residue. Firstly, the char residue of MIL-PET was deposited on the upper side at initial combustion, which was exposed to the higher oxide concentration and constitute a rough exterior layer (Figure 9e). With the catalyzed thermal decomposition at present of ferric ions in MIL-88B(Fe), a smooth, fragile, and relatively dense interior layer (Figure 9d) was formed which was similar to the char residue of PET, P-PET, and MIL-P-PET at 600 °C. In conclusion, plenty of oxygen, effective catalysis centers (herein ferric ions), high temperature above its DTG peak value, and sufficient combustion time were the key factors to the generation of high-quality char residue of flame-retardant PET.

### 3.6. Flame-Retardant Mechanism

**Figure 10:**
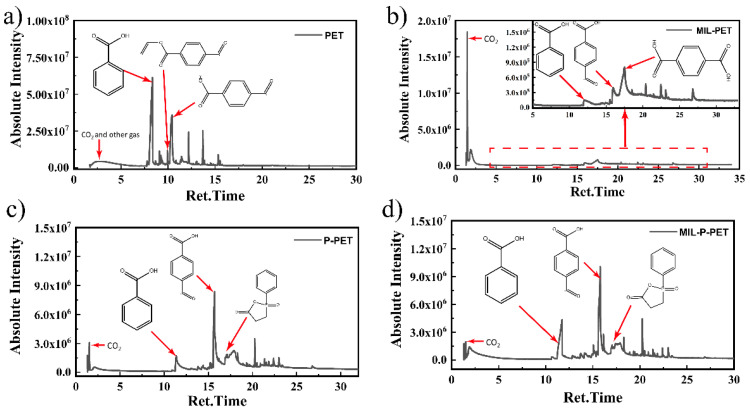
Py-GC-MS spectra of (**a**) PET, (**b**) MIL-PET, (**c**) P-PET, (**d**) MIL-P-PET.

Py-GC-MS is one of the most efficient analysis methods to detect the thermal decomposition behavior of MIL-PET and MIL-P-PET. As seen in Figure 10, compared with pure PET, MIL-PET produced more CO_2_, illustrating that the ferric ions in MIL-88B(Fe) catalyzed the thermal decomposition of PET and produced more gases. After zooming in on Figure 10b, the peak of vinyl 4-formylbenzoate was too minute to be detected, though terephthalic acid appeared and became the superior product. It demonstrated that the generation of terephthalic acid replaced the vital process of 4-formylbenzoate formation and extinction after MIL-88B(Fe) was added to the PET. However, the prolysis products of MIL-P-PET were similar to those of P-PET, proving that the ferric ions in MIL-88B(Fe) were inactivated by phosphorus flame retardant, i.e., CEPPA in the PET matrix. The peak amount of benzoic acid increased, which indicated a restricted fire resistance for CEPPA. Therefore, it seems as though MIL-88B(Fe) coordinated with the decomposition products of CEPPA.

**Figure 11:**
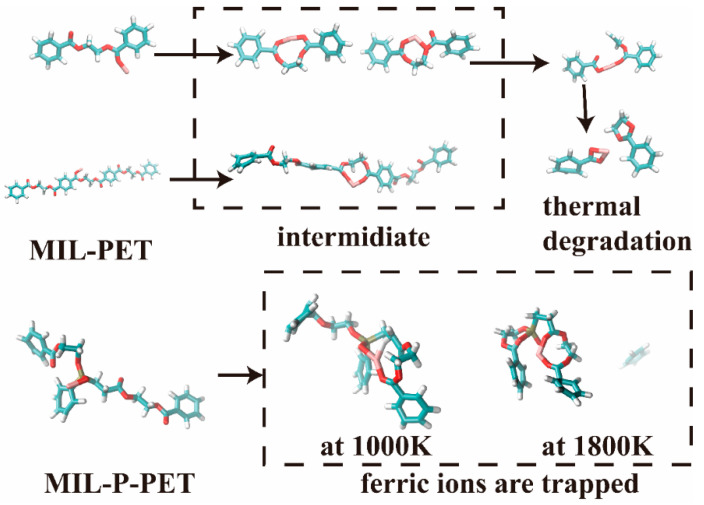
The representative video clips in quantum chemistry molecular dynamics of MIL-PET and MIL-P-PET at 727 °C (1000 K) and 1527 °C (1800 K).

Semi-empirical quantum chemistry molecular dynamics could be one of the most effective ways of studying thermal degradation of the polymer matric at the molecular level in the future [33]. GFN1-xTB is one of the cheapest functions used to calculate the metal complex and simple polymer unit movement, as well as thermal degradation at picosecond level. It can be seen in Figure 11 that ferric ions generated an intermediate, and then degraded as a ferric terephthalate complex in an N_2_ atmosphere at 727 °C (1000 K) within 104.75 ps in the simplified model. After increasing the molecular weight, it became harder to degrade within 400 ps but ferric was still activating. By contrast, ferric ions coordinated with P=O and C=O in P-PET were becoming difficult to degrade at 727 °C (1000 K) within 400 ps. This structure was still stable even at a higher temperature of 1527 °C (1800 K). This might be mainly due to the bond angle of pentavalent phosphorus, which promoted the possibility of complexing ferric ions with multiple C=O bonds. These results were in accordance with previous Py-GC-MS results.

**Figure 12:**
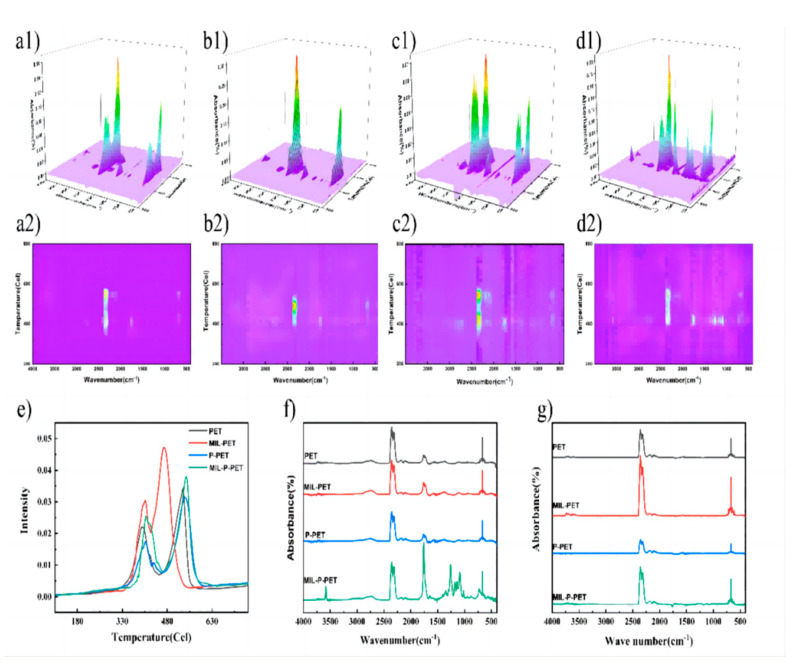
3D spectra and its vertical view of TG-FTIR in air atmosphere of (**a1**,**a2**) PET, (**b1**,**b2**) MIL-PET, (**c1**,**c2**) P-PET, and (**d1**,**d2**) MIL--P-PET. The (**e**) Gram-Schmidt curves and (**f**,**g**) FTIR in the second highest and highest peaks of Gram-Schmidt curves of PET, MIL-PET, P-PET, and MIL-P-PET.

Further, TG-IR in the air atmosphere could be a more visual means of illustrating the thermal oxide decomposition product. The 3D spectra and its vertical view could intuitively describe the thermal degradation process, and the results are displayed in Figure 12 and Figure S6. This further confirmed that PET and its composites had two thermal degradation steps and a shorter degradation progress was observed in MIL-PET (Figure 12a–d). The Gram-Schmidt curves in Figure 12e well disclosed the concentration changes of the gas-phase. This suggested that the first step of degradation would be violent, resulting in noticeable mass loss and heat release, but lower gas-phase concentration. The second peaks in the Gram-Schmidt curves of MIL-PET were advanced, meaning that ferric ions were mainly catalyzing the gas generation instead of smoke in the first step.

Moreover, the peak at 3750 cm^−1^ (Figure S6, which strongly correlated with the water vapor) of the second thermal decomposition step was more substantial than that of the first peak. This confirmed that dehydrogenation was more likely to happen in the second step to generate water vapor. Compared with other PET composites, the ratio value of the peak in the second and first step of thermal decomposition (*A*_f_/*A*_s_) for MIL-PET was 4.912, which was much higher than that of the Gram-Schmidt curves, which was 1.549. This result could prove the occurrence dehydrogenation reaction of MIL-PET, which mainly happened in the second step and produced much more water. This also explained the reason why the C-H-stretched vibration of benzene char residue in FTIR of char residue for MIL-PET disappeared at 500 °C. The results of the Gram-Schmidt curves further proved the negligible effect MIL-88B(Fe) had on the thermal decomposition of P-PET. The value of *A*_f_/*A*_s_ showed a slight drop due to the phosphoric acid dehydration in the first step (Figure S6). However, this influence could not be observed in MIL-P-PET, indicating that the coordination product of phosphate and ferric ions invalided its dehydration capability.

The FTIR spectra of PET and its composites are shown in Figure 12f,g to illustrate the gas-phase composition in two steps thermal oxide decomposition. CO_2_ (2319, 2352, and 669 cm^−1^), CO (2196 and 2094 cm^−1^), and acetaldehyde (2759, 1760, 1351, and 1116 cm^−1^) were detected in PET, MIL-PET, P-PET, and MIL-P-PET. The most exciting finding was that the FTIR adsorption peaks of the first step gaseous product for MIL-P-PET was quite different from the characteristic absorption of the benzene ring (900–1400, 730, and 3075 cm^−1^) and carboxyl groups (1758 and 3581 cm^−1^). These results suggested that more benzoic acid was generated in air after combining MIL-88B(Fe) with the P-PET, which was regarded as the main resource of smoke and received an increased SPR in CC test. CO_2_ production in MIL-PET was much higher than CO production, suggesting that MIL promoted the conversion of CO to CO_2_ and explained the reduced CO amount in the CC test.

TG-IR in nitrogen atmosphere was also analyzed, and the results are shown in Figure S7. The adsorption peak of benzoic acid was hard to determine for P-PET, demonstrating that CEPPA hindered PET decomposition from generating small molecules and producing more smoke than PET and MIL-PET, as seen in the CC test. The change in C=O intensity happened in PET and P-PET in different atmospheres, but did not for MIL-P-PET. It was assumed that ferric ions in the phosphate complex, which had a sizeable steric hindrance, preventing oxygen from attacking CH_2_ reaction sites after coordinating with the C=O. At the same time, because of the high steric hindrance, for the iron phosphate complex, it was hard to generate a stable intermediate like the free ferric ions in MIL-PET to change the whole mechanism.

### 3.7. Thermal Degradation and Carbonization Mechanism

**Figure 13:**
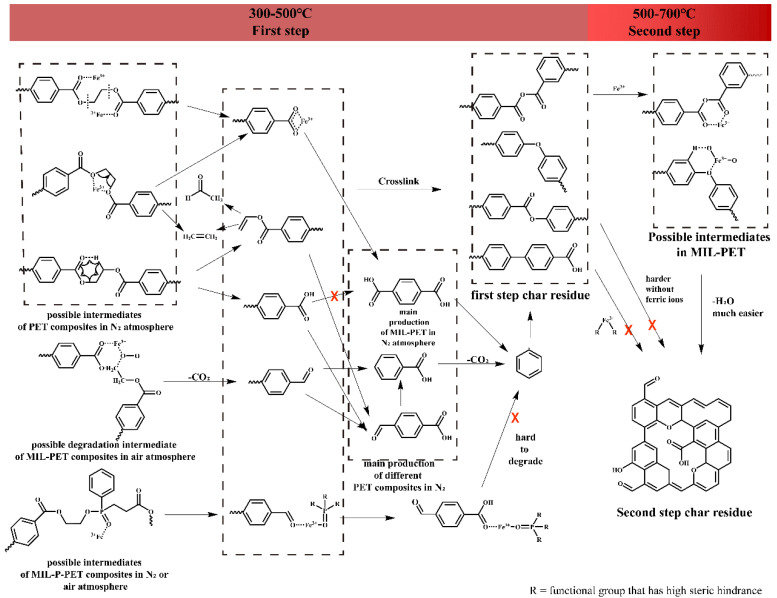
Possible thermal degradation mechanism for MIL-PET.

As seen in Figure 13, the thermal degradation of MIL-PET and MIL-P-PET proceeded in a two-step decomposition. In an N_2_ atmosphere, the ferric ions in MIL-88B(Fe) coordinated with the C=O bond to catalyze to the homolytic cleavage of PET, causing a large amount of terephthalic acid generation for MIL-PET, while the production of benzoic acid and 4-formylbenzoic-acid were dominant in PET due to the competition between homolytic cleavage and the β-cis-elimination reaction [48]. However, in air atmosphere, the oxygen, which has been proven to influence the CH_2_ bond in previous research [49], accelerated the degradation or cross-linking of terephthalic acid and benzoic acid. In MIL-P-PET, the ferric ions generated a stable ferric phosphate complex, which possessed quite a few empty coordinate orbits to coordinate with the oxygen atoms in the PET, and the phosphate ligands exhibited a high steric hindrance, inhibiting the decomposition of PET and resulting in heavy smoke and the loss of the catalysis to second step char residue generation.

**Figure 14:**
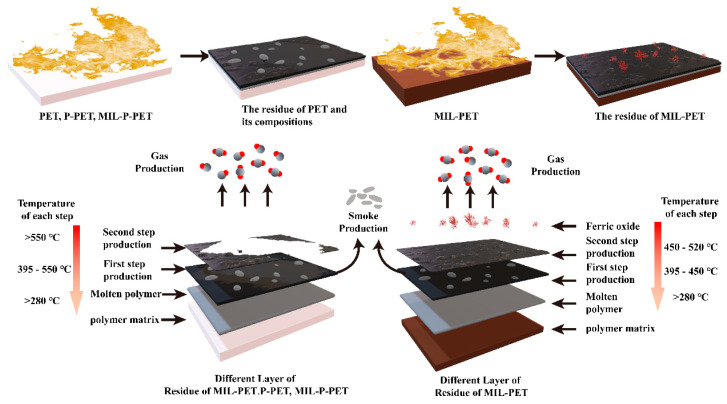
The possible carbonization mechanism for MIL-PET and MIL-P-PET.

The possible carbonization mechanism of MIL-PET is shown in Figure 14. It was assumed that PET and its composite generated several char layers after the two steps of thermal oxide decomposition. For PET, P-PET, and MIL-P-PET, the matrix of polymer mainly proceeds first step reaction to form a stable and smooth char residue with trace exterior char layer, while the char residual was composed of both a rougher exterior layer containing ferric oxide, and a dense inner layer for MIL-PET, owing to the high efficient ferric ion catalysis in MIL-88B(Fe). Moreover, a large amount of CO_2_ was generated in a short time on the surface to dilute oxygen concentration. In detail, the phosphorus in P-PET played the role of dehydration carbonization, and MIL-BB(Fe) in MIL-PET was dominated by the catalytic carbonization. However, the carbonization was restricted due to coordinated complex between phosphorus and ferric ions.

## 4. Conclusions

In this study, MIL-88B(Fe) was blended into PET and P-PET to improve their flame-retardant properties. A LOI value of 27% and a pHRR reduction of 23.12% was achieved in 1.0MIL-PET. With the addition of MIL-88B(Fe), the generation of ferric ions containing intermediates had a significant catalytic effect on the thermal oxide degradation of PET. Ferric ions coordinating with oxygen atoms of the PET matrix accelerated the polymer decomposition and dehydrogenation to generate more CO_2_ and water vapor in the gas phase, while hierarchical char residual composed of a dense inner layer and a rough exterior layer with ferric oxides formed in condensed phase. For MIL-P-PET, the LOI value kept increasing to 30% and reached a V-0 rating, coupled with a 21.75% pHRR decrease. However, an unexpected phenomenon was observed in MIL-P-PET combustion. The catalysis ability of ferric ions in MIL-88(Fe) was inactivated in the presence of CEPPA in PET main chains, resulting in incomplete carbonization, to form dense char residual and sufficient CO_2_ and an increased smoke release. This was attributed to the quenching effect after the P=O and ferric ion complex during the combustion, which was also in accordance with the semi-empirical quantum chemistry molecular dynamics simulation. Therefore, the interaction effect between MOF and phosphorus-containing flame retardant should be primarily considered in the synergistic flame-retardant system design.

## Figures and Tables

**Table 1:** The recipes of MIL-88B(Fe) and PET composites.

Samples	PTA (phr)	EG (phr)	MIL-88B(Fe) (phr)	CEPPA (phr)
PET	100	44.86	0	0
0.2-MIL-PET	100	44.86	0.2	0
0.6-MIL-PET	100	44.86	0.6	0
1.0-MIL-PET	100	44.86	1.0	0
P-PET	100	44.86	0	4.5
0.2-MIL-P-PET	100	44.86	0.2	4.5
0.6-MIL-P-PET	100	44.86	0.6	4.5

**Table 2:** TG and DTG data of MIL-PET and MIL-P-PET composites.

Sample	N_2_ Atmosphere			Air Atmosphere		
	*T*_5%_/°C	*T*_max_/°C	Char/*wt*%	*T*_5%_/°C	*T*_max_/°C	Char/*wt*%
PET	395.54	434.95	12.60	362.31	437.22	0.00
0.2MIL-PET	399.03	439.46	18.55	381.03	431.95	0.00
0.6MIL-PET	394.35	436.92	15.17	388.61	429.22	0.00
P-PET	395.43	442.79	12.09	338.75	434.52	4.77
0.2MIL-P-PET	386.15	442.52	8.44	362.48	438.34	0.19
0.6MIL-P-PET	382.37	439.56	9.38	377.37	437.50	0.00

**Table 3:** Results of LOI and VFT tests for MIL-PET and MIL-P-PET composites.

Sample	LOI (%)	UL-94 Test			
		*t*_1_(s)	*t*_2_(s)	Ignition of Cotton	Rate
PET	22	32.6	25.8	YES	NR
0.2MIL-PET	25	4.4	2.7	YES	V-2
0.6MIL-PET	26	3.5	2.5	YES	V-2
1.0MIL-PET	27	2.3	2.3	YES	V-2
P-PET	29	1.8	1.9	YES	V-2
0.6MIL-P-PET	30	2.4	1.4	NO	V-0

Note: *t*_1_, *t*_2_: average time after the first and second ignition.

**Table 4:** CC results of MIL-PET and MIL-P-PET composites.

Sample	PHRR (KW/m^2^)	THR (MJ/m^2^)	TSP (m^2^)	PCOP (g/s)	pCO2P (g/s)
PET	800.07	49.86	24.62	0.02	0.56
0.2MIL-PET	811.49	57.79	29.51	0.02	0.64
0.6MIL-PET	721.53	53.91	23.77	0.01	0.56
1.0MIL-PET	615.87	60.10	24.35	0.01	0.45
P-PET	839.30	50.20	29.97	0.05	0.60
0.6MIL-P-PET	656.79	49.83	51.02	0.03	0.48

## Data Availability

The data presented in this study are available on request from the corresponding author. The data are not publicly available due to current security.

## Supplementary Materials

The following supporting information can be downloaded at: https://www.mdpi.com/article/10.3390/polym15010224/s1. Figure S1: (a) Temperature changes vs. combustion time of MIL-PET in LOI test and (b) its measurement setup; Figure S2: SEM image (a) and element mapping images (b–d) for 0.6 MIL-PET; Figure S3: The micro cone calorimeter curves of PET, MIL-PET, P-PET, MIL-P-PET; Figure S4: C1s and O1s of X-ray photoelectron spectroscopy and its fitted lines of char residue of PET (a1–a2), MIL-PET (b1–b2), P-PET (c1–c2), MIL-P-PET (d1–d2); Figure S5: Optical microscope of PET (a1–a2), MIL-PET (b1–b2), P-PET (c1–c2) and MIL-P-PET (d1–d2) at 500 and 600 °C; Figure S6: FTIR spetra at 3750 cm^−1^ of PET, MIL-PET, P-PET and MIL-P-PET at different temperature; Figure S7: The TG-FTIR spectra of the highest concentration of PET, MIL-PET, P-PET, and MIL-P-PET in N_2_ atmosphere (a) and the contrast between the first step degradation in air and highest concentration in N_2_ atmosphere of MIL-PET (b) and MIL-P-PET (c); Table S1: Raman results of PET, 0.2MIL-PET, 0.6MIL-PET, P-PET, 0.6MIL-P-PET.
