# Supplementary material for: Thermal Degradation and Carbonization Mechanism of Fe−Based Metal−Organic Frameworks onto Flame−Retardant Polyethylene Terephthalate
Source: Polymers (Basel). 2023 Jan 1;15(1):224. doi: 10.3390/polym15010224 (PMC9823990; doi:10.3390/polym15010224)
Supplement: Supplementary file 1 [file polymers-15-00224-s001.zip › polymers-2105589-supplementary.pdf]

# Supporting information

## Thermal degradation and carbonization mechanism of Fe-based metal-organic frameworks onto flame retardant polyethylene terephthalate

**Tianyi Ma [1], Wenqing Wang [1,2,*], Rui Wang [1,2,*]**

[1] Materials Design & Engineering Department, Beijing Institute of Fashion Technology, Beijing 100029, China;
[2] Beijing Key Laboratory of Clothing Materials R&D and Assessment, Beijing Engineering Research Center of Textile Nanofiber, Beijing Institute of Fashion Technology, Beijing 100029, China;
* Correspondence: Wenqing Wang, 20180021@bift.edu.cn; Rui Wang, clywangrui@bift.edu.cn

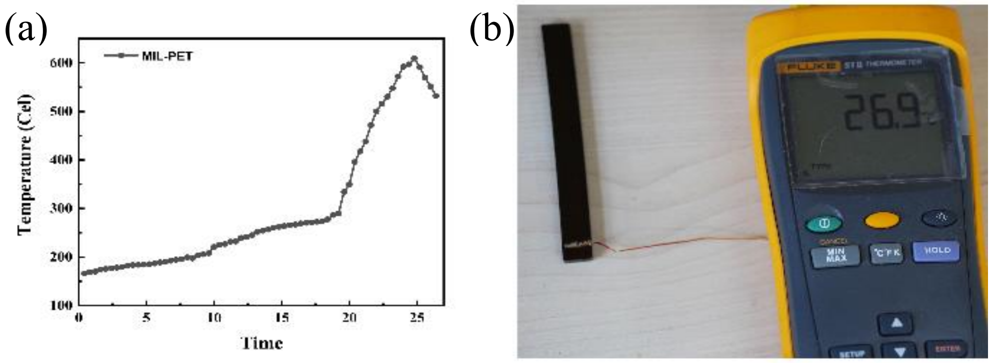


**Figure S1.** (a) Temperature changes vs. combustion time of MIL-PET in LOI test and (b) its measurement setup

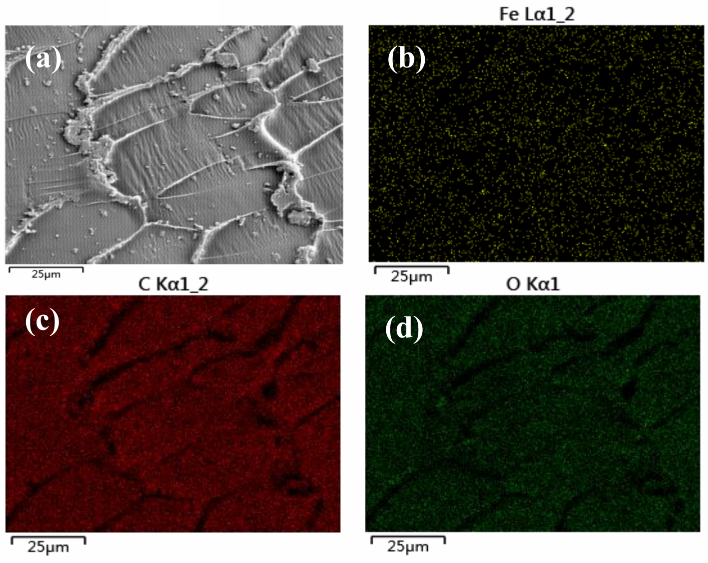


**Figure S2.** SEM image (a) and element mapping images (b-d) for 0.6 MIL-PET

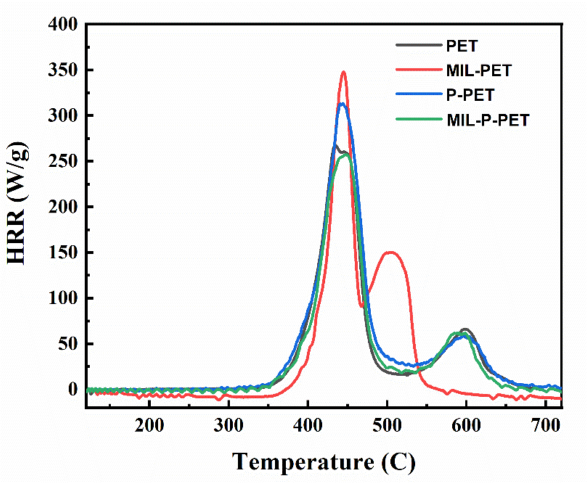


**Figure S3.** The micro cone calorimeter curves of PET, MIL-PET, P-PET, MIL-P-PET

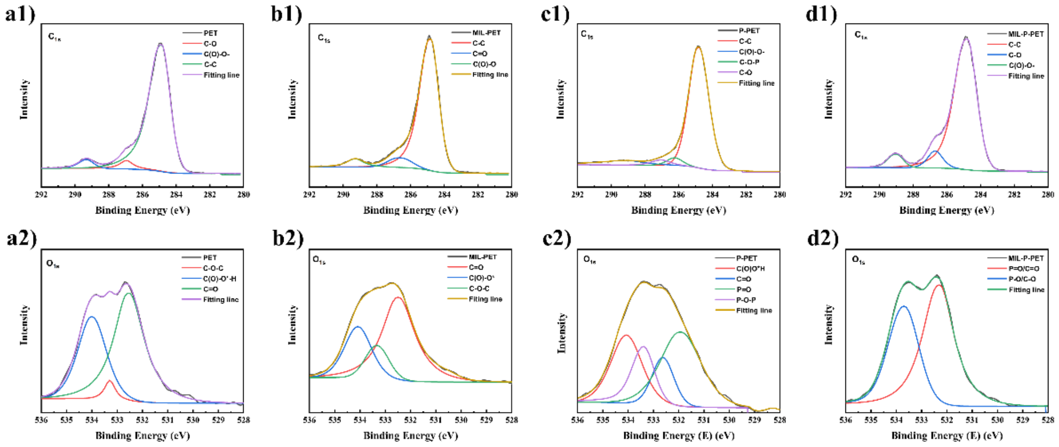


**Figure S4.** C1s and O1s of X-ray photoelectron spectroscopy and its fitted lines of char residue of PET a1-a2), MIL-PET b1-b2), P-PET c1-c2), MIL-P-PET d1-d2)

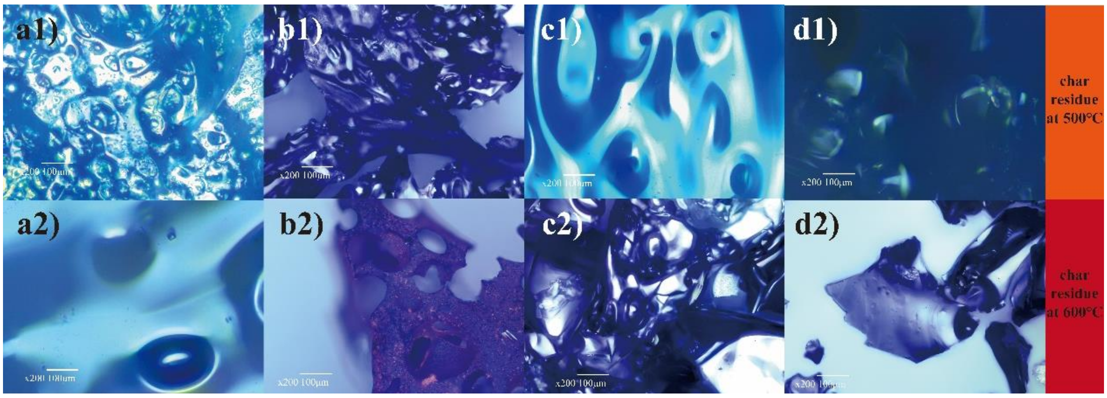


**Figure S5.** Optical microscope of PET a1-a2), MIL-PET b1-b2), P-PET c1-c2) and MIL-P-PET d1-d2) at 500 and 600 °C

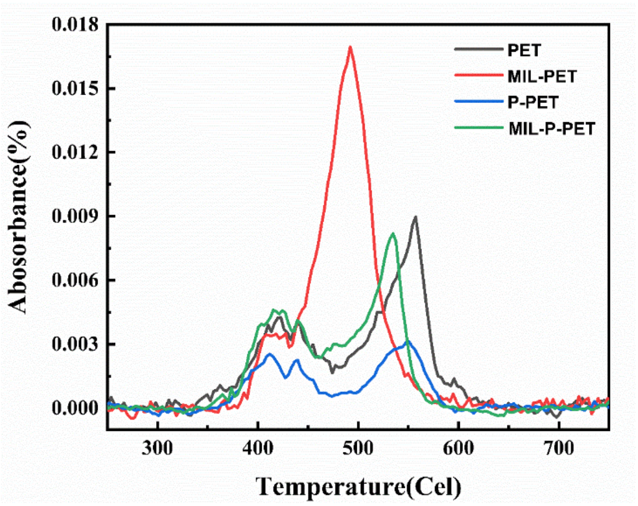


**Figure S6.** FTIR spetra at 3750 $cm^{-1}$ of PET, MIL-PET, P-PET and MIL-P-PET at different temperature.

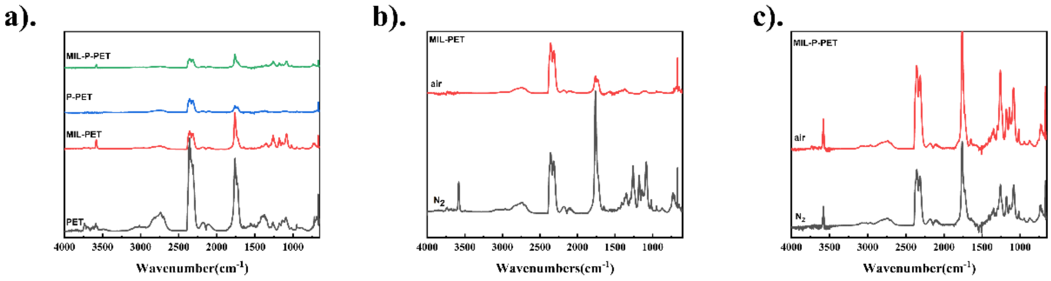


**Figure S7.** The TG-FTIR spectra of the highest concentration of PET, MIL-PET, P-PET, and MIL-P-PET in $N_2$ atmosphere (a). and the contrast between the first step degradation in air and highest concentration in $N_2$ atmosphere of MIL-PET (b)and MIL-P-PET (c).

**Table S1.** Raman results of PET, 0.2MIL-PET, 0.6MIL-PET, P-PET, 0.6MIL-P-PET

| Sample | $I_D$(×10$^5$) | $I_{D4}$(×10$^5$) | $I_{D3}$(×10$^5$) | $I_G$(×10$^5$) | $I_{D3}/I_G$ | $(I_{D3}+I_{D4})/I_G$ | $I_D/I_G$ |
|---|---|---|---|---|---|---|---|
| PET | 16.52 | 1.703 | 6.026 | 8.228 | 0.73 | 0.94 | 2.01 |
| MIL-PET | 9.232 | 1.827 | 3.820 | 6.064 | 0.63 | 0.93 | 1.52 |
| P-PET | 10.42 | 1.349 | 3.990 | 5.501 | 0.72 | 0.97 | 1.89 |
| MIL-P-PET | 7.024 | 1.046 | 3.21 | 4.588 | 0.70 | 0.93 | 1.53 |
